# The challenges of delivering addictions psychiatry teaching in the COVID era

**DOI:** 10.1192/bjo.2021.395

**Published:** 2021-06-18

**Authors:** Rebecca Hammersley, Amy Martin

**Affiliations:** Royal Edinburgh Hospital

## Abstract

**Aims:**

During the pandemic, addiction psychiatry moved from face-to-face lectures (delivered by Addictions Psychiatrists) to bitesize pre-recorded lectures (delivered by clinical teaching fellows) alongside interactive tutorials (delivered by Addictions Psychiatrists). The Addictions Team developed an online tutorial (delivered via Blackboard Collaborate) containing a combination of information slides, case studies, interactive quizzes, and short videos. These were delivered ‘live’ to small groups of students in up to four simultaneous virtual classrooms on a 6-weekly rolling basis. We aimed to assess student and tutor feedback regarding the move to interactive online tutorials in addiction psychiatry.

**Method:**

Two questionnaires sought feedback from students and tutors, focussing on the change from face-to-face to virtual teaching during a 20-week period.

**Result:**

21 (of 161) students completed the questionnaire.

- 100% ‘strongly agreed’ or ‘agreed’ that the content of the tutorial was relevant to learning outcomes.
- 52% felt ‘somewhat comfortable’ unmuting their microphones to contribute verbally, contrasting to 24% feeling ‘not very’ or ‘extremely uncomfortable’. In practice, only 30% of students contributed verbally.
- 57% felt most comfortable contributing via the written ‘chat’ function (rather than audio or camera).
- 65% felt either ‘somewhat’ or ‘very comfortable’ turning their cameras on, but only 1 student turned their camera on across all tutorials.
- 48% felt the tutorial was more accessible virtually than face-to-face; 5% considered it less so.
- When COVID restrictions are lifted, 14% would prefer the tutorial to remain virtual, 53% would rather it returned face-to-face, and 33% had no preference.

7 (of 7) tutors completed the questionnaire.

- 100% felt that students having their camera on would make their experience of delivering teaching ‘much better’ or ‘better’.
- 71% of tutors felt that students contributed ‘slightly’ or ‘significantly’ less in the virtual classroom.
- Only 29% of tutors found the experience of virtual teaching ‘very’ or ‘somewhat’ enjoyable, contrasting 43% finding it ‘somewhat’ or ‘very’ unenjoyable.
- Several white space tutor comments suggested the lack of audio-visual engagement made teaching less rewarding, whilst also preventing them from adapting content, pace, and teaching style to suit the group's needs. Tutors felt that the ease of delivering teaching from any location was beneficial.

**Conclusion:**

Virtual teaching has become embedded in medical education and will likely remain so post-pandemic. For it to be an effective and enjoyable experience, for both students and teachers, there needs to be adaptation of content, technology, etiquette and culture.

